# 3D DCT Based Image Compression Method for the Medical Endoscopic Application

**DOI:** 10.3390/s21051817

**Published:** 2021-03-05

**Authors:** Jiawen Xue, Li Yin, Zehua Lan, Mingzhu Long, Guolin Li, Zhihua Wang, Xiang Xie

**Affiliations:** 1 Institute of Microelectronics, Tsinghua University, Beijing 100084, China; xuejw18@mails.tsinghua.edu.cn (J.X.); yinl20@mails.tsinghua.edu.cn (L.Y.); lanzh18@mails.tsinghua.edu.cn (Z.L.); longmz14@mails.tsinghua.edu.cn (M.L.); zhihua@tsinghua.edu.cn (Z.W.); 2 Department of Electronic Engineering, Tsinghua University, Beijing 100084, China; guolinli@tsinghua.edu.cn; 3 The Graduate School at Shenzhen, Tsinghua University, Shenzhen 518055, China

**Keywords:** wireless capsule endoscopy (WCE), 3D DCT, image compression

## Abstract

This paper proposes a novel 3D discrete cosine transform (DCT) based image compression method for medical endoscopic applications. Due to the high correlation among color components of wireless capsule endoscopy (WCE) images, the original 2D Bayer data pattern is reconstructed into a new 3D data pattern, and 3D DCT is adopted to compress the 3D data for high compression ratio and high quality. For the low computational complexity of 3D-DCT, an optimized 4-point DCT butterfly structure without multiplication operation is proposed. Due to the unique characteristics of the 3D data pattern, the quantization and zigzag scan are ameliorated. To further improve the visual quality of decompressed images, a frequency-domain filter is proposed to eliminate the blocking artifacts adaptively. Experiments show that our method attains an average compression ratio (CR) of 22.94:1 with the peak signal to noise ratio (PSNR) of 40.73 dB, which outperforms state-of-the-art methods.

## 1. Introduction

**Figure 1:**
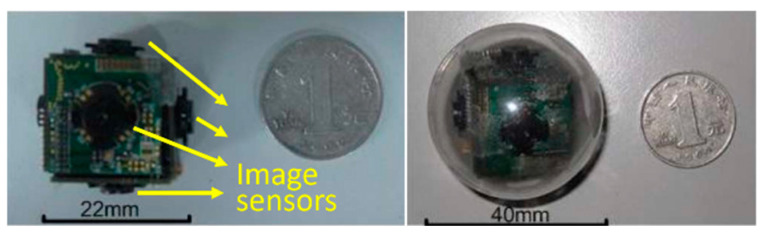
Prototype of the multiple cameras endoscopic capsule (MCEC), the print circuit boar (PCB) cube with six image sensors, is sealed into a transparent biocompatible plastic shell for practical verification.

Nowadays, in gastrointestinal (GI) tract diagnosis, the wireless capsule endoscopy (WCE) plays a vital role by enabling the visualization of the small intestine without any pain [1]. Once the capsule is swallowed by a patient, it begins to capture the GI tract by an inbuilt Complementary Metal Oxide Semiconductor (CMOS) camera. During its movement from mouth to anus, the captured images are transmitted wirelessly to an external image receiver system worn by the patient. Given the limited visual field, the low image acquisition rate, and the random movement of the WCE system, high missing rates occur inevitably in such devices. To address this issue, many research groups and companies exploited various technologies, for example, the multiple cameras endoscopic capsule (MCEC) system in the previous work of our group [2]. As illustrated in Figure 1, multiple image sensors are employed to get a larger visual field.

However, this solution leads to more image data and power consumption. Efficient image compression can be adopted here to reduce the wireless image transmitting power and save the wireless communication bandwidth [3]. It is noted that the high quality of compressed images has to be assured in this medical application.

In the WCE application, many compression algorithms have been proposed. Generally, they can be classified into two categories, i.e., lossless/near-lossless compression methods [4,5,6,7,8,9,10,11], and lossy compression methods [12,13,14,15,16,17,18,19]. In the early research on the compression for this medical application, to assure the quality of medical images, the former methods were generally adopted. JPEG-LS is favored in lossless/near-lossless approaches [4,5,6] as its low complexity, and high lossless compression performance with the compression ratio (CR) ≤ 4:1 and peak signal to noise ratio (PSNR) varies from 46.37 dB to ∞. To improve the lossless/near-lossless compression performance, a differential pulse code modulation (DPCM) followed with Golomb-Rice encoding is adopted [7,8,9,10,11]. In these methods, color-space transformations and subsampling schemes are generally incorporated as pre-processes to improve the coding performance with CR ≥ 4.5:1 and PSNR ≥ 48.2 dB. Although the lossless/near-lossless techniques assure the extremely high image quality, the compression ratio (≤5:1) cannot be improved anymore.

To improve the compression ratio while assuring acceptable image quality (PSNR about 40 dB), lossy compression methods have been discussed in many studies [12,13,14,15,16,17,18,19]. Some conventional color-space transformations, such as Y-U-V [12], Y-Cb-Cr [13], and Y-Cg-Co [14], are introduced before discrete cosine transform (DCT) to improve the compression performance, because they can decorrelate the color components to some extent. To find a more suitable color space transform of decorrelating the color components (CSTDCC), Reference [15] introduced the Y-E-F transform by analyzing the unique properties of WCE images to concentrate the energy to the Y channel. However, all the above transformations are not optimum CSTDCC. Thus, based on the Karhunen–Loève transform (KLT) theory, our team proposed Y-D1-D2-E color-space transformation [16], which is an optimum CSTDCC in a statistical sense. However, we found that the KLT based transform is only optimum statistically, instead of being the best transformation for each image or a set of images (e.g., one patient’s endoscopic image set). To reduce the power consumption of the image compression inside the capsule, there are also some works that focus on its low-complexity implementation [17,18,19], such as integer DCT followed by a quantization table composed of multiples of 2 [17].

In this paper, a 3D-DCT based compression method is proposed. We smartly use DCT to decorrelate the color components instead of KLT by reconstructing the 2D Bayer data into 3D blocks. Considering low complexity implementation, as well as high precision, we also propose a 4-point DCT butterfly structure without multiplication operation. By analyzing the unique characteristics of the data processed by 3D DCT, an improved meta-heuristic algorithm is leveraged to get a suitable quantization table, and a new 3D zigzag order is introduced for better coding performance. After decompression, we eliminate the annoying blocking artifacts by a frequency-domain approach.

The rest of this paper is organized as follows. The analysis of endoscopic images is discussed in Section 2. Then the 3-D DCT compression method and the deblocking algorithm are introduced in Section 3. Section 4 shows the experimental results. Conclusions are drawn in Section 5.

## 2. Two Properties of Endoscopic Images

**Figure 2:**
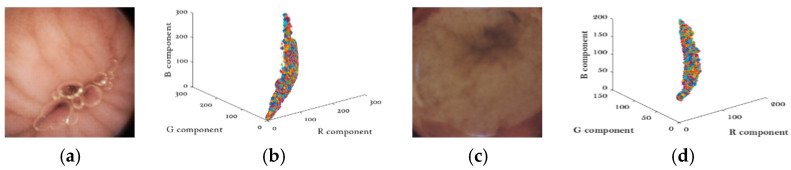
(**a**,**c**) Typical wireless capsule endoscopy (WCE) images (**b**,**d**) Color components distributions.

Unlike natural images, WCE images are much smoother and appear to be pink or yellow in general [16]. Another important property of WCE images is that WCE images have strong correlations between color components. As shown in Figure 2. Two typical WCE images are depicted in Figure 2a,c, and their pixel intensities are plotted in R-G-B space in Figure 2b,d. The almost-line shape distribution in the R-G-B space indicates the strong correlations between the three color-components.

To further verify our conjecture, the correlation coefficient *ρ* is adopted for quantifying the correlation. Table 1 gives the statistical results by testing more than 30,000 WCE images. The correlation coefficients of all combinations are higher than 0.9, which validates the property. In this paper, our method is designed to take full advantage of the two properties; the details will be discussed in Section 2 and Section 4.

## 3. The Proposed 3D DCT Compression Method

**Figure 3:**
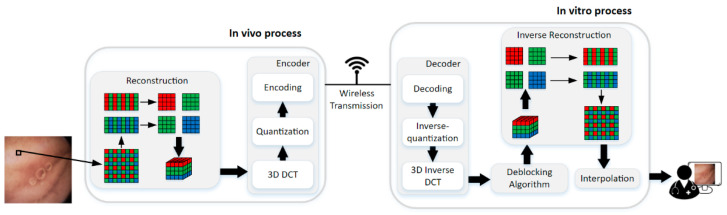
The framework of the proposed compression method.

The framework of the proposed method is illustrated in Figure 3. The method can be divided into two parts, i.e., in vivo process and in vitro process. The encoder is in vivo to compress the WCE image data with raw Bayer pattern, and it contains 3D-DCT, quantization, and encoding steps. Decoder and the deblocking algorithm in vitro are for the good visual effect of the reconstructed data. The decoder is the inverse of the encoder, so the 3D inverse DCT, inverse-quantization, and decoding steps are the inverse process of 3D DCT, quantization, and encoding, respectively, which will not be discussed in this paper anymore. Finally, the deblocked Bayer pattern data are interpolated into full-color images for diagnosis. The details will be discussed in the following paragraphs.

### 3.1. 3D Block Construction

**Figure 4:**
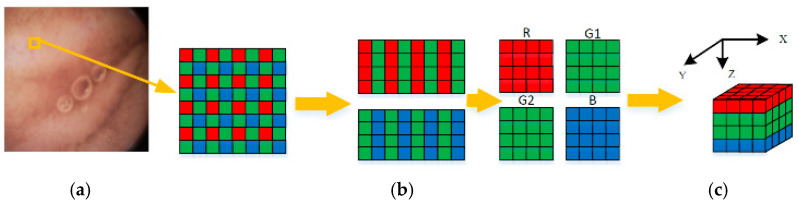
Construction of 3D blocks (**a**) Raw Bayer pattern (**b**) Regrouped color components (**c**) 3D blocks.

For low power and small size, capsule endoscopies generally adopt the CMOS image sensor. The Bayer pattern, as illustrated in Figure 4a, is widely used for a color filter array (CFA) of such sensors [20]. Different from the RGB image format, the Bayer pattern has only one channel, and the proportion of G, B, and R components is 2:1:1. Therefore, interpolation is required when the Bayer pattern is converted to a full-color image. From the compression viewpoint, the interpolation operations inevitably introduce redundancies before compression. Thus, using the Bayer pattern data directly in compression procedures is a better choice.

To remove the correlations among three color components efficiently, a Bayer pattern data has been reconstructed into several 3D blocks, as illustrated in Figure 4. Firstly, the Bayer pattern image is divided into 8 × 8 blocks. Then, three color components in a block are regrouped independently into three blocks. Due to the fact that the data amount of the G component is twice times the other two components, the G component of odd and even rows are divided into G1 and G2 blocks, respectively, as shown in Figure 4b. According to Table 1, both *ρ* (G,B) and *ρ* (G,R) are higher than *ρ* (R,B). Thus, the four blocks are stacked into a 4 × 4 × 4 3D block in the order of R-G1-G2-B, as shown in Figure 4c. This construction of 3D blocks makes the energy more concentrated at low frequencies, and the constructed 3D blocks can be directly applied to 3D-DCT to remove not only the correlations in three color components, but also the correlations among them. The reason why we choose 4 × 4 × 4 instead of other sizes is that, on the one hand, the ‘cube’ structure is easier to process; on the other hand, this size is more computation-friendly. For instance, the 4 × 4 × 4 blocks in this paper require only 9.75 additions and 3 shifts per pixel (as discussed in the next part), while the 8 × 8 × 4 blocks require 12.75 additions and five shifts per pixel. Therefore, the size of 4 × 4 × 4 is chosen to assure low computational complexity of 3D-DCT, as well as high precision.

### 3.2. 3D DCT Based Block Transformation

**Figure 5:**
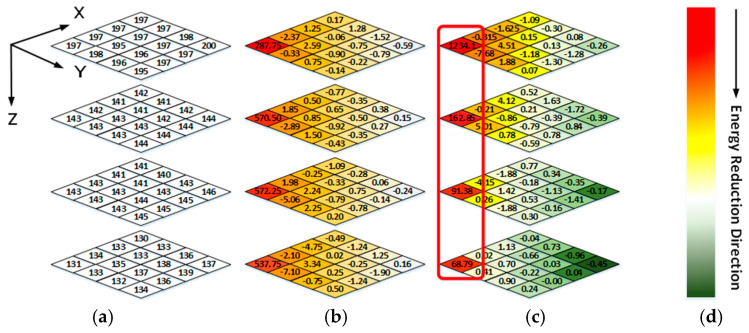
(**a**) Raw data of one image block (**b**) The frequency spectrum data of 2-D DCT (**c**) The frequency spectrum data of 3-D DCT (**d**) Color bar of energy intensity representation.

Figure 5a shows a 4 × 4 × 4 data block from a WCE image. The data intensities of R-G1-G2-B generally have a unique relationship, i.e., R > G1 ≈ G2 > B, because pink and yellow are the dominant hues of WCE images (discussed in Sec II). A 3D coordinate is established, as shown in Figure 5, where the X-O-Y plane coincides with the R block plane, and the R-G1-G2-B blocks are stacked along the Z direction. The frequency spectrum of each color block plane can be obtained by applying 2D DCT in the X-Y direction. As shown in Figure 5b, for a point (*x*, *y*, *z*) in z_th_ block plane (*z* = 0, 1, 2, 3), if *x* = *y* = 0, it is a DC component of the frequency spectrum in *z_th_* block plane. The corresponding frequency changes from low frequency to high frequency as x and y increase. After 1D DCTs are applied along the Z direction, the correlations among R, G1, G2, and B can be reduced greatly, as shown in Figure 5c.

Thus, a 4 × 4 × 4 DCT is adopted here, as illustrated in Formula (1). Where *f* (*n_1_*,*n_2_*,*n_3_*) is an input image pixel at (*n_1_*,*n_2_*,*n_3_*), and *F*(*i*,*j*,*k*) stands for the corresponding frequency-domain coefficients at (*i*,*j*,*k*).

(1) $$\begin{array}{c} F\left(i,j,k\right)=\sqrt{\frac{1}{8}}C_{i}C_{j}C_{k}\overset{3}{\underset{n_{1}=0}{\sum }}\overset{3}{\underset{n_{2}=0}{\sum }}\overset{3}{\underset{n_{3}=0}{\sum }}f\left(n_{1},n_{2},n_{3}\right)A_{n_{1},i}A_{n_{2},j}A_{n_{3},k}\\ C_{i}=\{\begin{array}{l} \frac{1}{\sqrt{2}},i=0\\ 1,i=1,2,3 \end{array}\;A_{p,q}=\cos\frac{\left(2p+1\right)q\pi }{8} \end{array}$$

It is well-known that 4 × 4 × 4 DCT can be decomposed into 4-point DCT in X, Y, and Z directions, respectively. Here, the DCTs of X and Y directions are used to remove the correlations inside the color components, and the correlations among them are removed by the DCT of Z direction. To decrease the computational cost of the 4-point DCT, our group proposes an architecture in Reference [21] which only needs to multiply $\sqrt{2}$ once. Since the DCT compression process is in vivo, here this multiplication is further simplified. According to $\sqrt{2}\approx 1+2^{-1}-2^{-4}-2^{-5}+2^{-7}$, we replace the multiplication operation by four additions and four shifts with the precision loss less than 1.5 × 10^−4^. Therefore, the proposed 4-point DCT structure requires only 13 additions and 4 shifts, the low computational complexity of 3D-DCT, as well as high precision can be assured.

### 3.3. Quantization

After 3D-DCT, a 4 × 4 × 4 quantization table should be derived by the rate-distortion theory. According to the rate-distortion theory [17,22], the optimization objective function can be expressed by Formula (2).

(2) $$\mathrm{Objective}=\underset{\varrho }{\min}\left\{\mathrm{MSE}\left(Q\right)+\lambda BR\left(Q\right)\right\}$$

**Figure 6:**
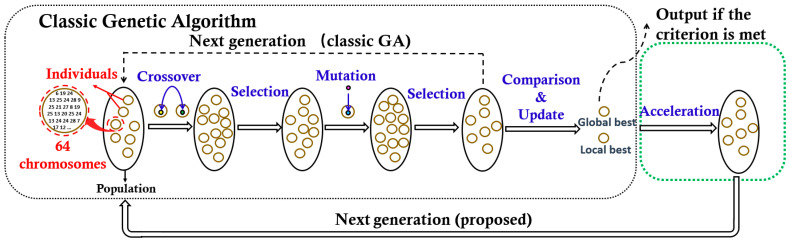
One generation procedure of the proposed algorithm (the arrow of the dotted line indicates the data flow direction of the genetic algorithm (GA) algorithm).

Formula (2) is formed by a weighted sum of the bit rate (BR) metric and the mean squared error (MSE) metric through the Lagrange multiplier *λ*. To implement the objective, we propose a hybrid meta-heuristic algorithm that combines the advantages of the genetic algorithm (GA) [23], and the particle swarm optimization (PSO) [24] jointly. As depicted in Figure 6, the proposed optimization framework consists of two parts, the classic GA algorithm, and the PSO based acceleration algorithm.

In the classic GA algorithm, the potential quantization tables are regarded as ‘individuals’ colored in yellow, 64 chromosomes in each individual correspond to 4 × 4 × 4 elements in a quantization table. A set of individuals donates as a ‘population’. The population is evolved through the iteration operation, which includes four steps, such as mutation, selection, crossover, and selection. Each iteration would generate a new evolved population as the next generation. The iteration is not stopped until it meets the maximum iteration number (500 iterations are needed in this paper). To guarantee a good global searchability, the individuals in a new generation are generated randomly, which also leads to a low search speed. To improve the search speed, while keeping the individual-diversity, we introduce a PSO based acceleration module to update the individuals closer to the potential optimal ones before the next generation initialized. Here, the acceleration Formula (3) based on the PSO theory [24,25] is adopted to update the individuals.

(3) $$d_{i+1}=wd_{i}+f\left(b_{i}-v_{i}\right)+f\left(g-v_{i}\right)$$

Here *b_i_* and *g* are the best individuals in the *ith* generation and all generations, respectively. *d_i_* represents the difference between the current individual *v_i_* and the optimal individual. To update *d_i_*, *w* is an inertia weight to describe the influence of the previous value, and *f*(*x*) = *kx* describes the influence of the difference between *v_i_* and *b_i_*, or *v_i_* and *g*. Then, the current individual is updated by *v_i+_*_1_ *= v_i_ + d_i+_*_1_. More details can be found in Reference [25].

### 3.4. Zigzag Scan

**Figure 7:**
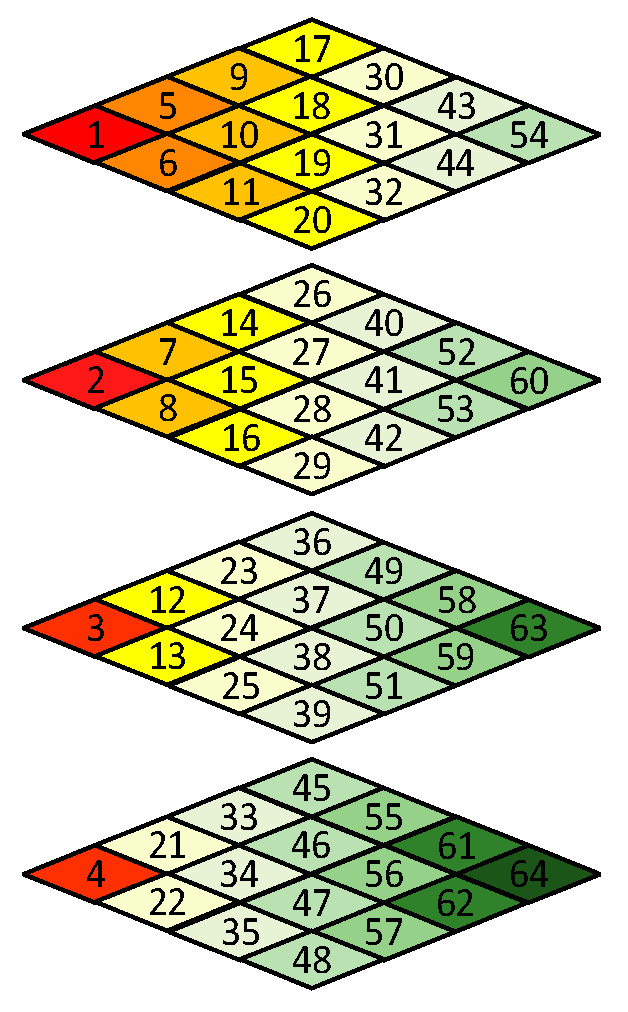
3D Zigzag scan order.

Like in other image or video compression methods, the scan order has to be decided before encoding. Since the frequency spectrum characteristics of 3D blocks are different from normal 3D video data [26], the scan order needs to be redesigned. Figure 5a shows a 4 × 4 × 4 data block from a WCE image. The data intensities of R-G1-G2-B generally have a unique relationship, i.e., R > G1 ≈ G2 > B, because pink and yellow are the dominant hues of WCE images (discussed in Sec II). As shown in Figure 5c, since points (0, 0, *z*) with *z* = 0, 1, 2, 3 contain DC components of all color planes, they are encoded by the DC component encoding rule like in JPEG standard [27]. For the remained AC frequency components, since the data in Figure 5b have both positive and negative values, the DCT transformed results would not be arranged in a specific order, i.e., from large to small. Therefore, a statistical analysis is carried out to determine the order. A subset of 321 WCE images was randomly selected from large amounts of data, resulting in 288,900 3D blocks in total. The scan order is determined by the statistical values of each frequency component, such as mean and variance. The larger the statistical value of the AC frequency component is, the earlier it is scanned, which can take advantage of long-run zeros. The final scan order is shown in Figure 7. Like the JPEG standard, run-length encoding followed with Huffman encoding is applied to the reordered AC coefficients.

### 3.5. Deblocking Algorithm

**Figure 8:**
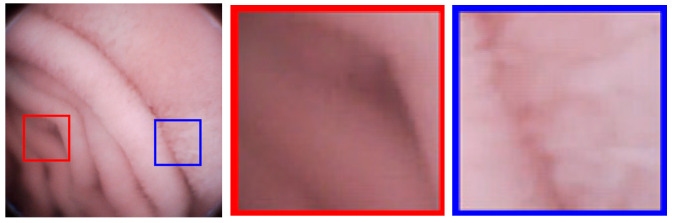
Blocking artifacts demonstration in an image with peak signal to noise ratio (PSNR) = 41.63 dB: Blocking artifacts are more perceptible in flat regions (red framed) than non-flat regions (blue framed).

Block-DCT usually brings blocking artifacts even when the objective image quality is high. As shown in Figure 8, an image with PSNR = 41.63 dB still has blocking artifacts; the artifacts are generally weak, but still perceptible to human eyes. For medical applications, image deblocking has to be adopted. It is known that image details are very important for medical diagnoses. However, in order to eliminate artifacts, the existing image deblocking algorithms may generally result in detail loss or blurry, which results from the fact that the algorithms can’t accurately judge whether it is a ‘real’ edge (tissue patterns) or a ‘false’ edge (blocking artifacts) at the block boundary. For examples, in spatial-domain deblocking methods, they generally utilize a threshold of intensity variation to make a decision on block boundaries [28,29], such as the mean squared difference of slope (MSDS), or the threshold is adaptive to the values of the quantization parameters like in the H.264/AVC and H.265/HEVC standards [30]. Unfortunately, the intensities of real edges could be any one, so it may lead to the real edges blurry or lost, and the false edge still remained. In frequency-domain deblocking methods, the transformed coefficients are modeled as a Laplacian distribution [31] or a Cauchy distribution [32], assuming that images are stationary. However, the assumption is too stringent. References [33,34] introduce the quantization error as uniformly distributed additive noise, and the non-local statistics are utilized to restore the original frequency coefficients to overcome non-stationary problem. All the above frequency-domain methods are based on statistical characteristics, which still makes it difficult to accurately judge the real or false edges. In this paper, a new evaluation criterion, the Sensitivity of MSDS (SMSDS), is proposed for blocking artifacts. According to the evaluation criterion, different smooth strategies are applied adaptively to remove the artifacts, while preserving the details of WCE images. The proposed deblocking algorithm is named, as the guided frequency-domain filter (GFF).

#### 3.5.1. The SMSDS Block Evaluation Criterion

**Figure 9:**
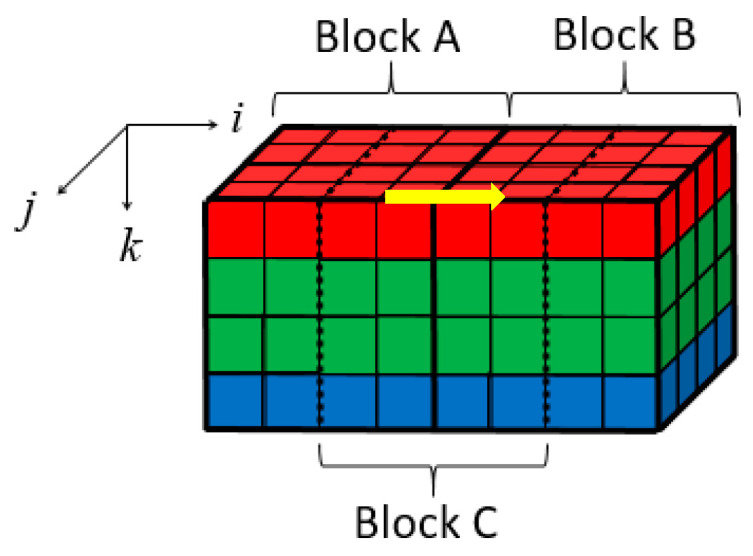
Location illustration of blocks A, B, and C.

In this method, the 4 × 4 × 4 blocks are directly demosaiced. As shown in Figure 9, a three-dimensional coordinate system is established for each 3D block. Its origin is the upper left corner pixel of the upper surface in each 3D block. Formula (4) illustrates the MSDS along the *i*-axis direction (the yellow arrow direction in Figure 9. Here *A*(*I*,*j*,*k*) and *B*(*I*,*j*,*k*) are spatial-domain matrices of blocks A and B.

(4) $${\mathrm{MSDS}}_{i}=\overset{3}{\underset{n_{2}=0}{\sum }}\overset{3}{\underset{n_{3}=0}{\sum }}[B\left(0,n_{2},n_{3}\right)-A\left(3,n_{2},n_{3}\right)-\frac{A\left(3,n_{2},n_{3}\right)-A\left(2,n_{2},n_{3}\right)+B\left(1,n_{2},n_{3}\right)-B\left(0,n_{2},n_{3}\right)}{2}{]}^{2}$$

From the above formula, it is known that the quantization errors and the image contexts at the boundary (real edges) may both lead to large MSDS values. Thus, MSDS is not good enough to distinguish between real edges and blocking artifacts. Fortunately, we found an interesting phenomenon that if a little dither ∆ is added to all the frequency coefficients, the MSDS values of blocking artifacts are more susceptible than that of the real edges. Based on this observation, we propose a new block evaluation criterion called the Sensitivity of MSDS (SMSDS) in the following formula.

(5) $$\mathrm{SMSDS}=\frac{\left|{\mathrm{MSDS}}_{F+\Delta }-{\mathrm{MSDS}}_{F}\right|}{{\mathrm{MSDS}}_{F}}$$

The subscript of the MSDS indicates whether it is calculated from the original spectrum *F* or the jittered spectrum *F +* ∆. In theory, if ∆ could compensate for the quantization distortion to some extent, for blocking artifacts, MSDS*_F+_*_∆_ can be regarded as the higher order of infinitesimal of MSDS*_F_*, which leads to SMSDS ≈ 1. For real edges, MSDS*_F+_*_∆_ ≈ MSDS*_F_* and SMSDS ≈ 0. Therefore, we adopt the artificially estimated quantization error model from [35] to define the amplitude of ∆ as

(6) $$\Delta_{ijk}=\frac{Q\left(i,j,k\right)}{2}\coth\left(-\frac{\xi \left(i,j,k\right)Q\left(i,j,k\right)}{2}\right)-\frac{1}{\xi \left(i,j,k\right)}$$

*Q*(*i*,*j*,*k*) is the quantization coefficient, *F*(*i*,*j*,*k*) is frequency components at (*i*,*j*,*k*) and ξ(*i*,*j*,*k*) = 5$\sqrt{2}$/*F*^2^(*i*,*j*,*k*). Thus, the SMSDS criterion is established by Formulas (5) and (6). The larger the SMSDS is, the stronger filtering is needed.

#### 3.5.2. The Guided Frequency-Domain Filter

As shown in Figure 9, let the right half of decompressed block A and the left half of decompressed block B form a new 4 × 4 × 4 block denoted as block C. Block C contains the boundary pixels and could be modified to eliminate blocking artifacts. The modification is expressed in the following formula [31]

(7) $$F_{c}^{\prime }\left(i,j,k\right)=\alpha_{i,j,k}F_{c}\left(i,j,k\right)+\beta_{i,j,k}\left[F_{A}\left(i,j,k\right)+F_{B}\left(i,j,k\right)\right],\;\alpha_{i,j,k}+2\beta_{i,j,k}=1$$

where *F_A_*(*i*,*j*,*k*), *F_B_*(*i*,*j*,*k*), *F_C_*(*i*,*j*,*k*) and *F_C_’*(*i*,*j*,*k*) are frequency components of blocks A, B, C and modified block C at (*i*,*j*,*k*), respectively. *F_A_* and *F_B_* can be obtained directly from the transmitted data, *F_C_* requires an additional 3D DCT from the decompressed data. *α_i_*_,*j*,*k*_ and *β_i_*_,*j*,*k*_ describe how much the previous component is retained and the guidance impact, respectively. Inspired by Reference [36], an objective function (8) is defined to restore the smoothness of block C, while preventing *F_C_’* change too much from *F_C_*, then solved by the linear ridge regression method as illustrated in Formula (9)

(8) $$Objective=\min\{[\alpha_{i,j,k}F_{C}\left(i,j,k\right)+\beta_{i,j,k}F_{A}\left(i,j,k\right){+\beta_{i,j,k}F_{B}\left(i,j,k\right)-F_{C}\left(i,j,k\right)]}^{2}+\varepsilon_{i,j,k}\alpha_{i,j,k}^{2}\}$$

(9) $$\begin{array}{c} \alpha_{i,j,k}=\frac{{\left(F_{c}\left(i,j,k\right)-\frac{F_{A}\left(i,j,k\right)+F_{B}\left(i,j,k\right)}{2}\right)}^{2}}{{\left(F_{c}\left(i,j,k\right)-\frac{F_{A}\left(i,j,k\right)+F_{B}\left(i,j,k\right)}{2}\right)}^{2}+\varepsilon_{i,j,k}} \end{array},\beta_{i,j,k}=\frac{1-\alpha_{i,j,k}}{2}$$

Here *ε_i_*_,*j*,*k*_ is a regularization parameter to penalize large *a_i_*_,*j*,*k*_. Its value is related to the predefined block evaluation criterion and decided by Formula (10)

(10) $$\varepsilon_{i,j,k}=SMSDS\cdot \eta Q\left(i,j,k\right)$$

In Formula (10), ηQ(i,j,k)  is utilized to evaluate the quantization degree. η = 0.5 represents the impact of quantization on the frequency spectrum. *Ε_i_*_,*j*,*k*_ is positively related to quantization degree *Q*(*I*, *j*, *k*), thus η  is positive.

## 4. Results

**Figure 10:**
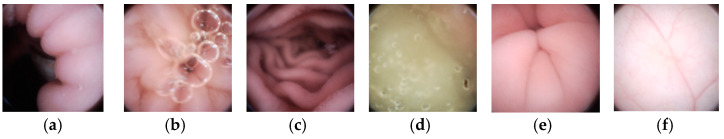
Six typical WCE images from the dataset (Interpolated from raw Bayer pattern) (**a**,**c**,**e**) have rich villi and folds, (**b**,**d**) have bubbles, (**f**) is full of vessels.

In this section, experiment results are presented to demonstrate the outstanding performance of our method. A WCE image dataset with 15,216 images was built from seven patients. Each image is with a full resolution of 480 × 480 and a Bayer pattern. The dataset includes all kinds of typical endoscopic images, such as villi, folds, blood vessels, air bubbles, and flat GI tract walls, as shown in Figure 10. Note that, images in Figure 10 are interpolated from their raw Bayer pattern data. In this paper, the proposed meta-heuristic algorithm is leveraged to get the optimal quantization table under different *λ* values. Given the limited power supply and bandwidth in WCE systems, we aim to use only one quantization table that can achieve the highest CR by ensuring the average PSNR of decompressed images is larger than 40 dB. The quantization we use here is shown in (11).

(11) $$Q\left(:,:,1\right)=\left[\begin{array}{cccc} 8 & 16 & 16 & 32\\ 8 & 32 & 32 & 32\\ 32 & 32 & 32 & 64\\ 16 & 16 & 32 & 64 \end{array}\right]Q\left(:,:,2\right)=\left[\begin{array}{cccc} 8 & 32 & 32 & 32\\ 32 & 32 & 32 & 32\\ 32 & 64 & 32 & 32\\ 64 & 64 & 32 & 64 \end{array}\right]\;Q\left(:,:,3\right)=\left[\begin{array}{cccc} 8 & 32 & 32 & 32\\ 32 & 64 & 32 & 32\\ 32 & 64 & 32 & 32\\ 64 & 64 & 64 & 32 \end{array}\right]Q\left(:,:,4\right)=\left[\begin{array}{cccc} 8 & 32 & 32 & 32\\ 32 & 32 & 32 & 32\\ 64 & 32 & 32 & 64\\ 32 & 32 & 64 & 32 \end{array}\right]$$

State-of-the-art algorithms with color-space transformations (e.g., Y-U-V [12], Y-Cb-Cr [13], Y-Cg-Co [14], Y-E-F [15], Y-D1-D2-E [16]) and without color-space transformation [17] are compared with our proposed method. The above methods are compared in three aspects: Objective/subjective qualities and computational complexity. The performances of objective quality include compression ratio (CR) and peak to noise ratio (PSNR). Some examples are exhibited to demonstrate the subjective visual image effect. At the end of this section, the computational complexity is assessed.

### 4.1. Objective Quality Comparison

**Figure 11:**
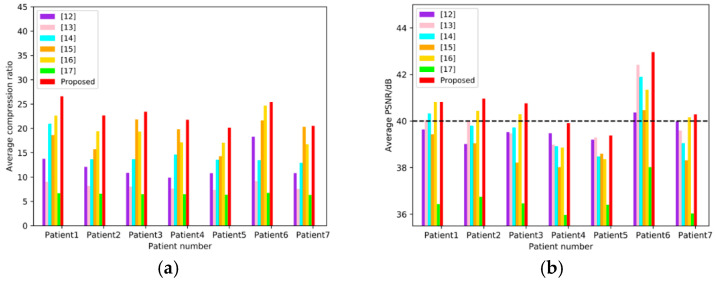
Objective evaluation base on 7 sub-datasets corresponding to 7 patients. (**a**) shows the average CR of each methods in the seven patients, and (**b**) shows the average PSNR of each methods in the seven patients, where the dotted line indicates PSNR = 40 dB.

To compare the objective qualities of the proposed method with those of References [12,13,14,15,16,17], the average CR and PSNR of each patient’s images are counted independently, and the results are illustrated in Figure 11. For each patient’s images, the metrics of the proposed method are larger than those of other methods. The average compression results of 7 patients are illustrated in Table 2. Reference [15] yields to relatively high average CR (>18:1), but relatively low PSNR (<39 B), and References [12,13,14] have high PSNR (>39 dB) with relatively low CR (<15:1). Reference [17] has an average PSNR less than 37 dB, as well as an average CR less than 7:1. The performance of Reference [16] can reach average CR > 19, as well as PSNR > 40 dB simultaneously, but ours is the best on both metrics (CR = 22.94:1 and PSNR = 40.73 dB).

### 4.2. Subjective Quality Comparison

**Figure 12:**
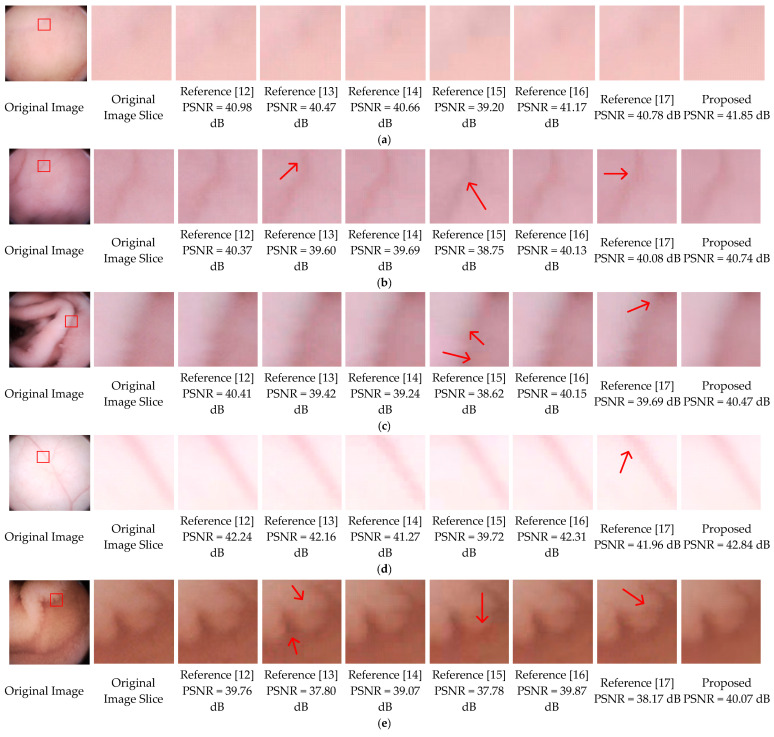
Final results comparison, where (**a**) is relatively flat, (**b**–**d**) have folds or vessels, (**e**) has rich villi.

In this part, the subjective image qualities of References [12,13,14,15,16,17], and the proposed method are compared. Figure 12 show typical endoscopic images. Figure 12a is relatively flat, (b,d) are images with folds or vessels, (e) has rich villi. Regions with a size of 70 × 70 are sliced from the original images to compare the different methods. As shown in the third, fifth, sixth, seventh columns of Figure 12, the decompressed images of References [12,14,15,16] are suffering from blocking artifacts. Although Reference [15] can achieve a compression ratio comparable to ours, its decompressed results have color distortions, especially in Figure 12b,e. To eliminate the annoying blocking artifacts, References [13,17], and the proposed method leverage different deblocking algorithms. Reference [13] adopts the adaptive deblocking filter (ADF) from H.264 standards, which calculates the intensity variations of the edges and classifies them using quantization-dependent thresholds. It works well in flat regions, such as the fourth columns of Figure 12a,c,d. However, the intensities of real edges in WCE images are ambiguous, some real edges with relatively low quantization degrees could be misjudged and blurred, for example, the upper area in the fourth column of Figure 12b. In the fourth columns of Figure 12e, some blocking artifacts in the villi areas are also misjudged and remained unchanged. In Reference [17], both the low frequency and high-frequency characteristics of adjacent blocks are considered, then a block evaluation criterion with fixed parameters is empirically derived. For the flat image in Figure 12a, the criterion performs well. Once the low frequencies and high frequencies of a block cannot meet the criterion, it will be left unsmoothed, such as the majority of the regions in the eighth columns of Figure 12b–e. In general, except for Figure 12a, the artifacts remain almost unchanged in all images. In this paper, the SMSDS block evaluation criterion is introduced. By assessing the susceptibility to frequency dither, real and false edges can be precisely distinguished. Then, a frequency-domain filter is applied to smooth the false edges adequately with preserving the real edges. As illustrated in the last columns of Figure 12a–e, the proposed deblocking algorithm works well in all situations.

**Figure 13:**
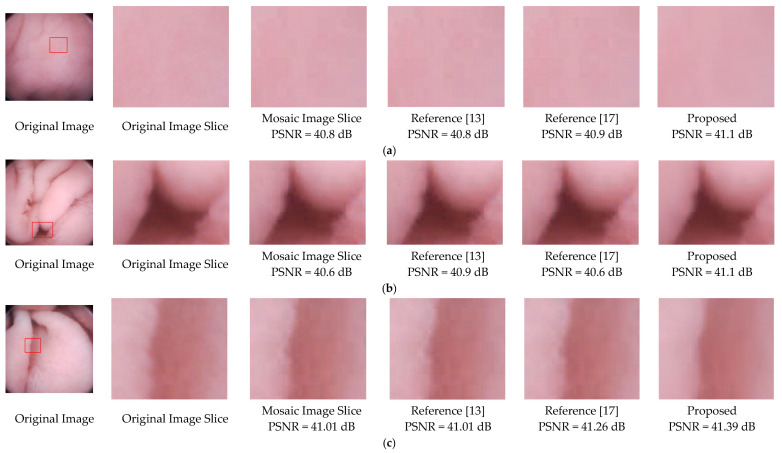
Deblocking performance comparison of References [13,17] and the proposed method. (**a**) is relatively flat, (**b**) are images with folds or vessels, (**c**) has rich villi. Note that regions with a size of 70 × 70 are sliced from the original images, and the methods were performed on the same mosaic images.

To further validate the effectiveness of the proposed deblocking method, we apply the methods of References [13,17], and the proposed to three mosaic images. As illustrated in Figure 13, blocking artifacts still exist in the results of References [13,17]. For instance, the edges of the folds in Figure 13b,c. Results of the proposed method not only have better visual effects, but also higher PSNR values.

### 4.3. Computational Complexity Comparison

In this part, the computational complexity comparison with state-of-the-art methods is carried out. Since References [15,16] uses 2n-based quantization tables like ours, they are selected for comparison. The computational cost of quantization and encoding steps can be considered as the same, thus only the color space transformations and DCT transformation steps are compared. Table 3 summarizes the numbers of addition and shift operations per pixel needed to perform the two steps in these methods. From this table, our proposed method reduces five shifts, but requiring 2.92 additions when compared to Reference [15].

Compared to Reference [16], the increased operations of addition and shift are 4.25 and 2, respectively. Meanwhile, in Table 2, values of CR and PSNR of the proposed are 21.39% and 4.79% higher than Reference [15], and 17.22% and 1.72% higher than Reference [16]. Although our method has higher computational complexity than previous works, it also improves the compression rate and visual image quality significantly. Power consumption is the dominant limitation of the proposed method. Still, given the limited bandwidth and power supplement in wireless endoscopy capsules, the proposed method can provide a broader prospect for further improving the frame rate of medical endoscopy image acquisition.

## 5. Conclusions

This paper had proposed a novel 3D DCT based compression method for medical endoscopic images. 3D pattern data are constructed from the raw Bayer pattern images. By taking the advantages of WCE image properties, the reconstructed images respond well to 3D DCT—therefore, the compression ratio can be significantly improved. The steps in the 3D compression process are ameliorated to lower the energy consumption and improve the coding performance. In the end, a frequency-guiding way of deblocking is utilized to compensate for the quantization distortion of the DCT method. Experimental results demonstrate that the proposed 3D compression method outperforms other state-of-the-art methods in terms of both subjective and objective image qualities, respectively.

## Figures and Tables

**Table 1:** Color components correlation coefficients of WCE images.

Correlation Coefficient	Average Value
ρ (R,G)	0.9575
ρ (R,B)	0.9041
ρ (B,G)	0.9526

**Table 2:** Comparison of state-of-the-art methods.

Method	Compression Type	Color Space	Algorithm	Deblocking	Average CR	Average PSNR
[12]	Lossy	Y-U-V	2D DCT	×	12.36:1	39.60 dB
[13]	Lossy	Y-Cb-Cr	2D DCT	√	8.14:1	39.97 dB
[14]	Lossy	Y-Cg-Co	2D DCT	×	14.67:1	39.74 dB
[15]	Lossy	Y-E-F	2D DCT	×	18.90:1	38.87 dB
[16]	Lossy	Y-D1-D2-E	2D DCT	×	19.57:1	40.04 dB
[17]	Lossy	/	2D DCT	√	6.53:1	36.58 dB
Proposed	Lossy	/	3D DCT	√	22.94:1	40.73 dB

**Table 3:** Computational complexity comparison (“a” for additions and “s” for shifts).

	[15]	[16]	Proposed
Color space transformation	2.33a + 3s	1.5a	/
DCT	4.5a + 5a	4a + 1s	9.75a + 3s
Total	6.83a + 8s	5.5a + 1s	9.75a + 3s

## Data Availability

No new data were created or analyzed in this study. Data sharing is not applicable to this article.
